# Using Primary Care Geographic Network Adequacy Metrics for VA Obstetric Referrals: a Foundation for Equitable, Timely, and Veteran-Centered Care

**DOI:** 10.1007/s11606-023-08191-7

**Published:** 2023-06-20

**Authors:** Kavita Vinekar, Karen Chu, Gracielle J. Tan, Nicholas J. Jackson, Donna L. Washington, Kristina M. Cordasco

**Affiliations:** 1 VA Health Services Research and Development Center for the Study of Healthcare Innovation, Implementation and Policy, Los Angeles, CA USA; 2 Division of Gynecology, Department of Surgery & Perioperative Services, VA Greater Los Angeles Healthcare System, Los Angeles, CA USA; 3 Department of Obstetrics & Gynecology, University of California Los Angeles (UCLA) David Geffen School of Medicine, Los Angeles, CA USA; 4 Division of General Internal Medicine and Health Services Research, Department of Medicine, University of California Los Angeles (UCLA) David Geffen School of Medicine, Los Angeles, CA USA

## INTRODUCTION

With rising numbers of reproductive-age women using Veterans Health Administration (VA) services, demand for maternity services has increased.^1^ Obstetric care is a VA benefit provided by contracted community providers.^2^ Like other Americans, Veterans suffer from high rates of severe maternal morbidity and stark racial inequities in maternal health outcomes.^3^ Geographic access to prenatal care is associated with lower risks of adverse pregnancy outcomes.^4^

VA classifies geographic network adequacy using drive time between the Veteran’s residence and VA-based or community-contracted care provider.^5^ This measure of network adequacy for all eligible Veterans is distinct from community care eligibility standards under the MISSION Act. Primary care networks are considered inadequate if drive time exceeds 30 min (45 min for rural Veterans) while specialty care networks are inadequate if drive time exceeds 45 min (100 min for rural Veterans). Current VA policy classifies obstetric care as specialty care. However, among maternal and public health experts, obstetric care is commonly viewed as primary care given its acuity and need for frequent visits.

We assessed the prevalence of inadequate geographic access to VA community obstetric care among women VA users residing in the Greater Los Angeles (GLA) catchment area, comparing use of primary versus specialty care designations.

## METHODS

Using the VA Provider Profile Management System (PPMS), a database of VA-contracted community providers, we identified all California network general obstetric providers as of 10/01/2021. Using ArcGIS Pro, we mapped these providers, overlaying a shapefile to identify those in the GLA catchment area, and conducted a systematic internet search to confirm they were actively practicing in California. Providers were excluded if they were not practicing obstetrics in California.

We identified reproductive-age (18–44 years) women Veteran VA enrollees with a valid address in the GLA catchment area as of 10/01/2021, using VA’s Corporate Data Warehouse, excluding those with missing urbanicity designations (45 of 8254 Veterans). We obtained Veteran age, race/ethnicity, urbanicity, and geocoded address. We mapped Veteran addresses and obstetric care sites in ArcGIS Pro and calculated nearest facility drive time. Geographic network adequacy was determined using both primary care and specialty care definitions*.* We compared Veterans with adequate versus inadequate obstetric care access, performing chi-square tests for categorical variables and Student’s *t*-tests for continuous variables, using Stata/MP v.17 (StataCorp, College Station, TX). The GLA Institutional Review Board approved this study.

## RESULTS

We identified 431 obstetric VA-contracted community providers, with 220 unique addresses, and 8209 women Veterans in the GLA catchment area. When using VA specialty care drive time targets, 84/8209 (1.0%) Veterans lacked adequate geographic access (Table 1). However, using VA primary care geographic access targets, 2480 (30.2%) had inadequate geographic access. Using a primary care designation, 31.3% of urban and 18.6% of rural Veterans lacked adequate geographic access to care (compared to 0.2% and 10.2% using specialty care designations, respectively). Veterans who identified as non-Hispanic White were more likely to have inadequate access (37.9% using a primary care designation), compared to others (range 22–29%) (Table 1).

**Table 1:** Adequate vs Inadequate Geographic Network Access to Nearest Obstetric Provider Among Veterans in Greater Los Angeles

	Total	Specialty drive times		*p*-value	Primary care drive times		*p*-value
		Adequate access	Inadequate access		Adequate access	Inadequate access	
Women Veterans ages 18–44	8209 (100)	8125 (99.0)	84 (1.0)		5729 (69.8)	2480 (30.2)	
Age, mean (SD)	34.8 (5.8)	34.8 (5.8)	33.8 (6.0)	0.10	34.7 (5.8)	35.1 (5.8)	0.007
Age groups, *n* (%)				0.05			0.07
18–24 yrs	397 (4.8)	392 (98.7)	5 (1.3)		278 (70.0)	119 (30.0)	
25–34 yrs	3321 (40.5)	3277 (98.7)	44 (1.3)		2363 (71.2)	958 (28.9)	
35–44 yrs	4491 (54.7)	4456 (99.2)	35 (0.8)		3088 (68.8)	1403 (31.2)	
Race/ethnicity, *n* (*%*)				< 0.001			< 0.001
American Indian/Alaska Native	55 (0.7)	53 (96.4)	2 (3.6)		40 (72.7)	15 (27.3)	
Asian/Pacific Islander	817 (10.0)	816 (99.9)	1 (0.1)		595 (72.8)	222 (27.2)	
Non-Hispanic Black	1198 (14.6)	1192 (99.5)	6 (0.5)		934 (78.0)	264 (22.0)	
Hispanic	2256 (27.5)	2244 (99.5)	12 (0.5)		1581 (70.1)	675 (29.9)	
Multi-race	163 (2.0)	162 (99.4)	1 (0.6)		120 (73.6)	43 (26.4)	
Non-Hispanic White	2330 (28.4)	2287 (98.2)	43 (1.9)		1446 (62.1)	884 (37.9)	
Unknown/decline	602 (7.3)	592 (98.3)	10 (1.7)		440 (73.1)	162 (26.9)	
Missing	788 (9.6)	799 (98.9)	9 (1.1)		573 (72.7)	215 (27.3)	
Urbanicity^‡^, *n* (*%*)				< 0.001			< 0.001
Urban	7495 (91.3)	7484 (99.9)	11 (0.1)		5148 (68.7)	2347 (31.3)	
Rural	714 (8.7)	641 (89.8)	73 (10.2)		581 (81.4)	133 (18.6)	

Comparing Specialty (Specialty Care Drive Time Access Designation Was Less Than 45 Min for Urban and Less Than 100 Min for Rural/Highly Rural) and Primary Care (Primary Care Drive Time Access Designation Was Less Than 30 Min for Urban and Less Than 45 Min for Rural/Highly Rural) Designations

^‡^Rural designation includes rural and highly rural (*n* = 17 Veterans were highly rural) and none had adequate access to an obstetric provider

## DISCUSSION

There was a marked difference in the proportion of Veterans classified as having adequate versus inadequate geographic access to obstetric care based on whether specialty or primary care drive time designations were used. Over 30% of our cohort lacked adequate geographic access to obstetric care when using primary care designations, compared to just 1% when classified as specialty care.

This study was limited by using PPMS, which may overestimate numbers of providers accepting VA patients. We did not include family medicine physicians since there were not specific designations available for obstetric care provision. Finally, we did not include information regarding Veterans’ parity or surgical history (including sterilization and hysterectomy). We were also limited by the availability of only mailing address geocodes, which may not always correspond to residential address. Future studies will include residential geocodes once these become available.

A shift to classifying obstetric care as primary care would be consistent with Medicaid access standards in many states.^6^ Further, given the frequency and acuity of obstetric care, using primary care rather than specialty drive time would be a more appropriate gauge of care accessibility and could guide VA in providing pregnant Veterans with access to more timely and Veteran-centered obstetric care.
